# Tongue Volume Influences Lowest Oxygen Saturation but Not Apnea-Hypopnea Index in Obstructive Sleep Apnea

**DOI:** 10.1371/journal.pone.0135796

**Published:** 2015-08-17

**Authors:** Sang Hyeon Ahn, Jinna Kim, Hyun Jin Min, Hyo Jin Chung, Jae Min Hong, Jeung-Gweon Lee, Chang-Hoon Kim, Hyung-Ju Cho

**Affiliations:** 1 Department of Otorhinolaryngology, Severance Hospital, Yonsei University College of Medicine, Seoul, Korea; 2 Department of Radiology, Severance Hospital, Yonsei University College of Medicine, Seoul, Korea; 3 Department of Otorhinolaryngology-Head and Neck Surgery, Chung-Ang University College of Medicine, Seoul, Korea; 4 The Airway Mucus Institute, Yonsei University College of Medicine, Seoul, Korea; Weill Cornell Medical College in Qatar, QATAR

## Abstract

**Objectives:**

The aim of this study was to identify correlations between sleep apnea severity and tongue volume or posterior airway space measured via three-dimensional reconstruction of volumetric computerized tomography (CT) images in patients with obstructive sleep apnea (OSA) for use in predicting OSA severity and in surgical treatment. We also assessed associations between tongue volume and Mallampati score.

**Methods:**

Snoring/OSA male patients (n = 64) who underwent polysomnography, cephalometry, and CT scans were enrolled in this retrospective study. OSA was diagnosed when the apnea-hypopnea index (AHI) was greater than 5 (mild 5–14; moderate 15–29; severe>30). The patients were also categorized into the normal-mild group (n = 22) and the moderate-severe group (n = 42). Using volumetric CT images with the three-dimensional reconstruction technique, the volume of the tongue, posterior airway space volume, and intra-mandibular space were measured. The volumes, polysomnographic parameters, and physical examination findings were compared, and independent factors that are related to OSA were analysed.

**Results:**

No associations between tongue volume or posterior airway space and the AHI were observed. However, multivariate linear analyses showed that tongue volume had significantly negative association with lowest O_2_ saturation (*r = 0*.*365*, *p = 0*.*027*). High BMI was related to an increase in tongue volume. Modified Mallampati scores showed borderline significant positive correlations with absolute tongue volume (*r = 0*.*251*, *p = 0*.*046*) and standardized tongue volume (absolute tongue volume / intramandibular area; *r = 0*.*266*, *p = 0*.*034*). Between the normal-mild and moderate-severe groups, absolute tongue volumes were not different, although the standardized tongue volume in the moderate-severe group was significantly higher.

**Conclusion:**

Absolute tongue volume showed stronger associations with lowest O_2_ saturation during sleep than with the severity of AHI. We also found that high BMI was a relevant factor for an increase in absolute tongue volume and modified Mallampati grading was a useful physical examination to predict tongue size.

## Introduction

Obstructive sleep apnea (OSA) is a common condition that is characterized by the collapse of the upper airway during sleep. Its prevalence is reported to be around 5%, and the prevalence of OSA without symptoms is reported to be up to 24%[1]. OSA is related to various diseases, such as cardiovascular[2], cerebrovascular[3], and endocrine diseases[4], or cognitive dysfunctions[5]. Therefore early diagnosis and treatment of OSA is very important. The diagnosis of OSA can be confirmed by polysomnography. However, OSA patients have a wide spectrum of symptoms and features that are influenced by sex, age, obesity, and anatomical factors. These diverse factors may influence surgical outcomes in various ways: Individuals with enlarged tonsils and a small tongue show higher surgical success rates. The most representative method for estimating tongue size is a modified Mallampati scoring method suggested by Friedman M et al.[6]. While the scoring method is an excellent predictor of surgical outcomes in OSA patients, the dimensions of the mandible can influence the palates position against tongue. A BMI greater than 40 is also a poor predictor of surgical outcomes [6], and obesity is significantly related to fat deposition in the posterior tongue [7]. Recently, obese rats and apneic patients exhibited a large degree of fat infiltration in the tongue [8, 9]. Therefore, meticulous physical examinations and evaluations of upper airway structures may help to choose optimal surgical procedures, especially tongue base resection to reduce tongue volume.

To predict the severity or obstruction level of OSA, various imaging techniques have been applied to quantitatively measure soft tissues or skeletal structures in the oral cavity. Previous cephalometric analyses have suggested that OSA patients exhibit an enlarged soft palate, decreased upper airway width at multiple levels, an inferiorly positioned hyoid bone, and the inferior shift of the enlarged tongue[10–12]. However, these analyses are limited by the fact that they present only two-dimensional (2-D) images[13]. With the recent development of various imaging techniques, computerized tomography (CT) or magnetic resonance (MR) imaging with three-dimensional (3-D) reconstruction has also been applied to analyze OSA patients[14, 15]. MR imaging has been shown to reveal close relationships between the volumetric data of oral cavity structures and the severity of sleep apnea, and these images demonstrate excellent soft tissue contrast[14]. However, MR imaging is susceptible to artifacts that can result from dentures or air in the oral cavity. Meanwhile, the advent of the state-of-the-art multidetector-row CT (MDCT) can provide high-quality images with fast acquisition times and reduce motion-induced image degradation. In addition, the image reconstruction procedure may be feasible in the evaluation of OSA patients, as it has become very easy and fast when used in daily practice. Among various measurements, performing 3-D analyses of space or volume of the upper airway are essential, because most sleep surgeries aim to widen the upper airway by resecting soft tissues or adjusting the position of the bony structure. Therefore, estimating the volume of the tongue or upper airway space may allow sleep surgeons to choose proper surgical methods with better outcomes that are tailored to the individual features of OSA patients. Some studies have shown correlations between upper airway volume and polysomnographic parameters [16–20]. However, variable results have also been reported, according to body mass index (BMI), sex, age, or race [16, 19–22]. There is also insufficient evidence showing a relationship between tongue volume and physical examination findings.

In this study, we aimed to reveal the relationships between tongue or airway space volume, as measured from 3-D-reconstructed MDCT scans, and polysomnographic parameters in OSA patients to provide useful insights of surgical treatment for OSA patients. Further statistical analyses were employed to investigate the influence of the size of the upper airway structure on sleep studies and physical examination findings to determine whether tongue volume can be estimated by the modified Mallampati staging method.

## Materials and Methods

### Inclusion criteria

We retrospectively reviewed the medical records of patients who visited the sleep clinic at the Department of Otorhinolaryngology at Yonsei University Severance Hospital from January 2012 to May 2013. Consents to assess and utilize their medical records for medical research was obtained from all patients. This retrospective study used medical records from patients whom were anonymized and de-identified just prior to when we accessed their medical records. This study was approved by the Institutional Review Board at Yonsei University College of Medicine (4–2014–0283). Data from Korean male patients who underwent overnight polysomnography, lateral cephalometry, and paranasal sinus CT were analysed. Sixty-four subjects (from 20 to 71 years old, mean age: 43.8 ± 12.3 years) were analysed. All patients underwent in-laboratory polysomnography. Respiratory events were classified according to the criteria defined by the American Academy of Sleep Medicine in 2007 [23]. Various parameters, such as apnea-hypopnea index (AHI), mean O_2_ saturation, lowest O_2_ saturation, respiratory disturbance index (RDI), and oxygen desaturation index (ODI) were also analysed. The AHI was calculated as the sum of the total events of the apnea index and hypopnea index. Apnea was defined as the absence of airflow for 10 seconds or longer. Hypopnea was defined as reduced airflow for at least 50% of the measured duration, with the presence of either oxygen desaturation ≥4% of the normal level or an arousal. The RDI was the sum of the AHI and respiratory effort-related arousal (RERA). The ODI was defined as the number of times per hour of sleep that the blood’s oxygen level dropped by 3% or more from baseline.

### Physical examination

The pharyngeal examination was performed to evaluate tonsillar hypertrophy, tongue size, and Friedman stage. Tonsil sizes were graded as follows [24]: Grade 1 = tonsils in the tonsillar fossa that were barely visible behind the anterior pillar; Grade 2 = tonsils were visible behind the anterior pillar; Grade 3 = tonsils extended three quarters of the way to the midline; and Grade 4 = tonsils completely obstructed the airway. To estimate tongue size, a modified Mallampati grading method was used as previously reported [24], because it is utilized in Friedman staging to predict surgical outcomes. The patient was asked to open his/her mouth widely with the tongue positioned in place three times, and oropharyngeal crowding was scored according to which sections of the mouth were clearly visible: Grade I = tonsils, pillars, and the soft palate; Grade II = uvula, pillars, and the upper pole; Grade III = tonsils, pillars, and part of the soft palate, but not the uvula base; and Grade IV = hard palate only. Friedman stages were graded as follows [6]: Stage I = palate position I or II, tonsil size 3 or 4, and BMI < 40; Stage II = palate position I or II with tonsil size 0, 1, or 2, or palate position III and IV with tonsil size 3 or 4 and BMI < 40; Stage III = palate position III or IV with tonsil size 0, 1, or 2 and all patients with a BMI > 40.

### Cephalometric evaluation

Standardized lateral cephalometric radiographs of the head and neck were analysed. The mandibular plane (line from the midpoint of the mandibular angle to the lowest point on the outline of the mentonian symphysis)-to-hyoid distance (MP-H) and posterior airway space (PAS, distance between the base of the tongue and the posterior pharyngeal wall) were measured and utilized for analyses, because these are related to tongue size and upper airway volume, respectively. These measurements were compared with volumetric measurements or polysomnographic parameters.

### CT acquisition and measurement

**Fig 1:**
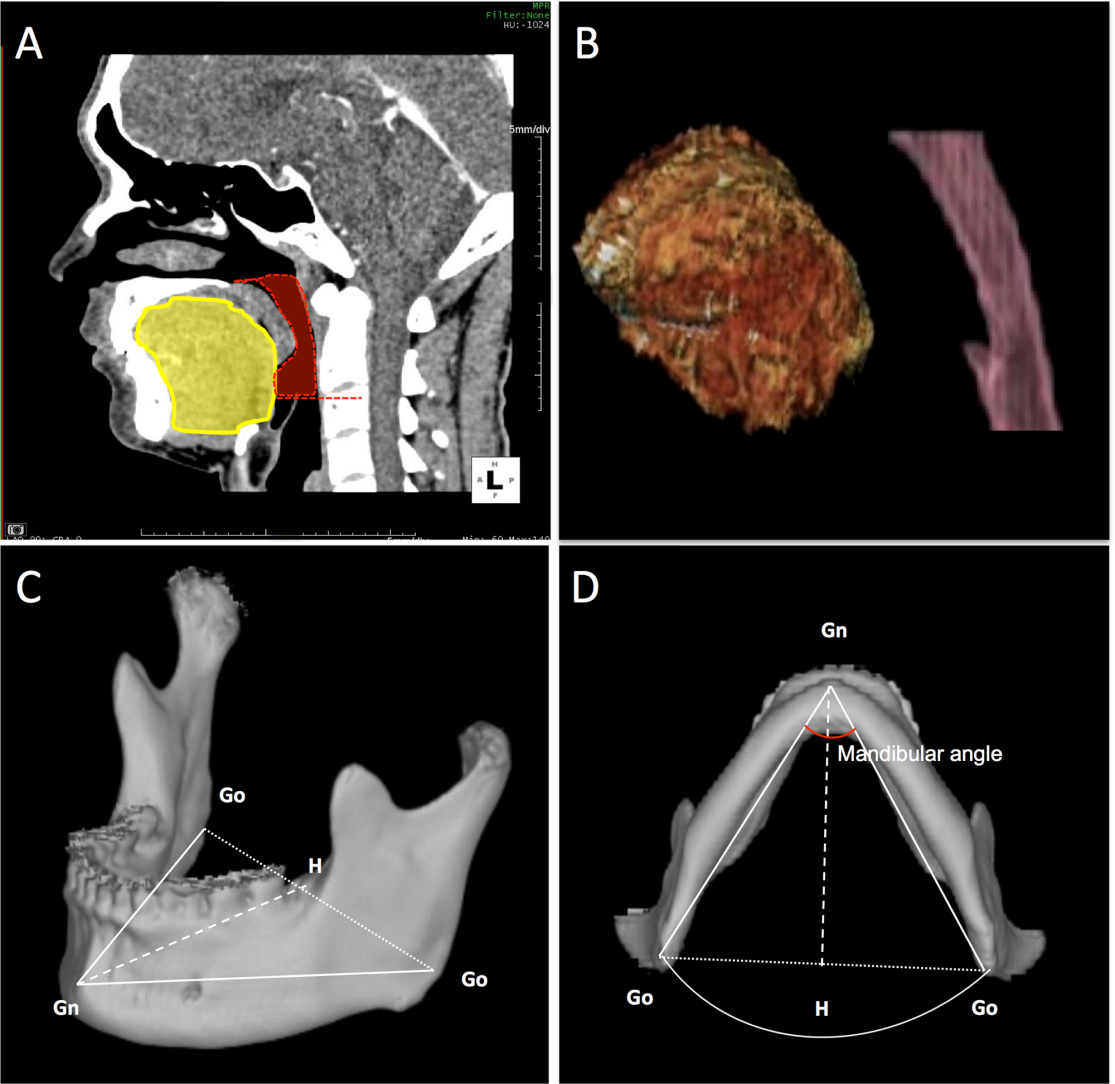
Analysis of three-dimensional (3-D)-reconstructed computer tomography (CT) images. A. The tongue (yellow) and posterior airway space (red) are seen on a sagittal reformatted image. B. Three-dimensional segmentation of the tongue and posterior airway space. C. Oblique view of the reconstructed mandible, which is marked with measured points. D. Inferior view of the reconstructed mandible showing intra-mandibular area. Gn; gnathion, Go; gonion, H; midpoint between both gonions, Gn-H; mandibular depth.

**Fig 2:**
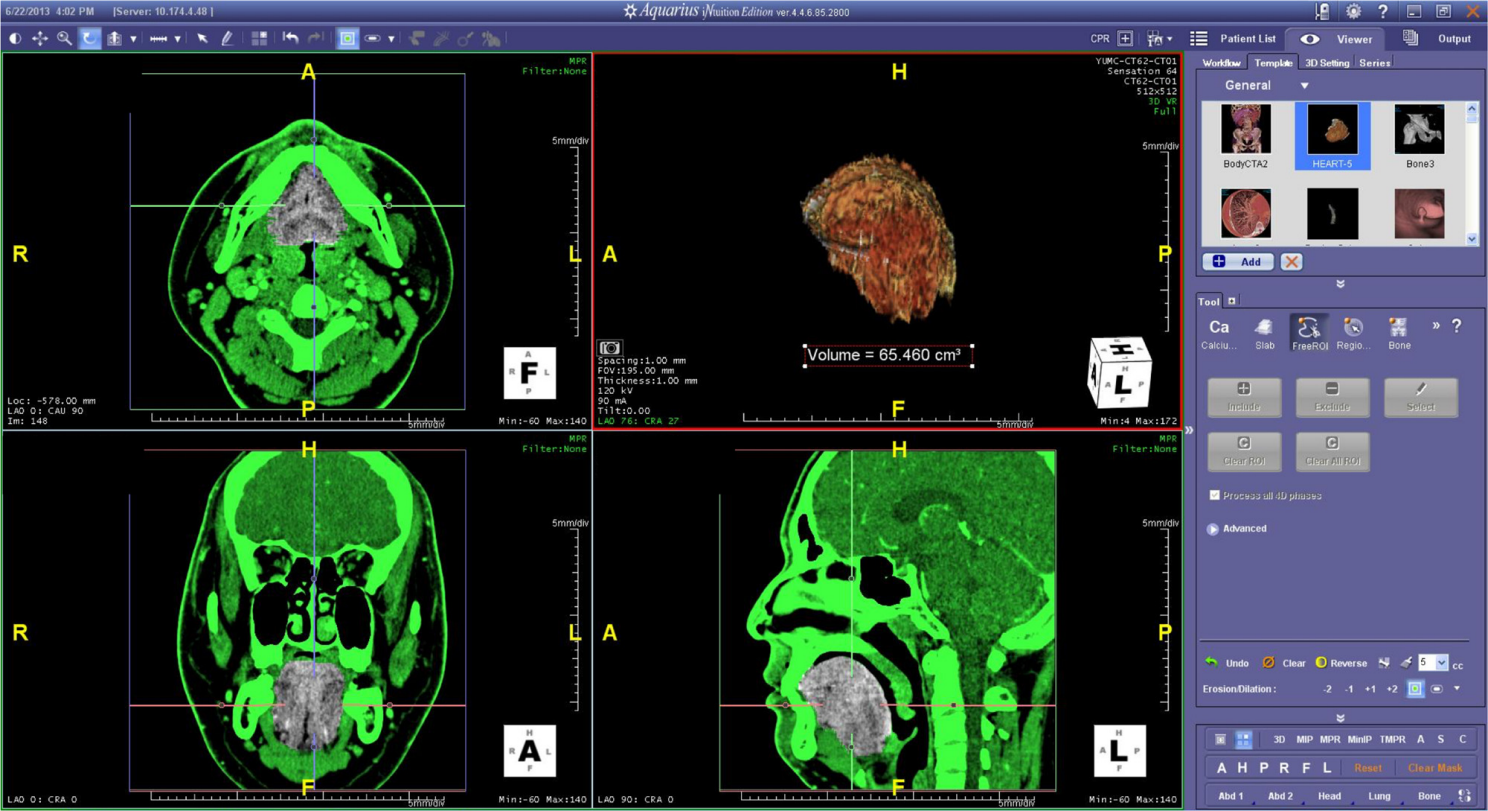
An example of 3D-reconstructed CT image of tongue and volume measurement by 3-D workstation software (Aquarius iNtuition).

Non-contrast CT examinations were performed with one of two CT scanners (Somatom Sensation 16 or 64; Siemens, Erlangen, Germany), using a standard CT protocol for the paranasal sinuses. All MDCT scans were taken in the supine position with the mouth closed, and scans were obtained in an average of multiple breaths. Contiguous CT images of the paranasal sinuses were obtained with a collimation of 0.6- or 0.75-mm from the top of the frontal sinus to the lower margin of the mandible, and reconstructed at 3-mm increments in the axial plane. Coronal and sagittal reformation of the CT images was also performed at 2- and 3-mm increments. Tube voltage and mA were 120 kVp and 200 mA, respectively. By using a dedicated 3-D workstation and software (Aquarius iNtuition; TeraRecon, San Mateo, CA), segmentations of the tongue, PAS, and mandible were performed to measure volumes (Figs 1 and 2). One researcher performed all measurements three times and averaged values were used for the analysis. The researcher was blinded to the subjects’ polysomnographic results. The outlines of the tongue were defined on all continuous sections of coronal images because it could clearly differentiate tongue margin from adjacent structures. All coronal-sectional areas were automatically summed up from the tongue base to the tongue apex by the software program (Fig 2). The anatomy of tongue structure was based on a previous report [16, 18, 21], and it included all intrinsic and extrinsic muscles (*i*.*e*., genioglossus, hypoglossus, and styloglossus), with the exception of muscles that line the floor of the mouth (*i*.*e*., digastric, mylohyoid, and geniohyoid).

Measurements of PAS volume (PASV) were performed as previously described [16, 25]. The posterior nasal spine was designated as the superior border of the upper airway, and the perpendicular plane from the posterior nasal spine represented the anterior border. The inferior border was the apex of the epiglottis, the posterior pharyngeal wall was the posterior border, and the lateral pharyngeal wall was the lateral border (Fig 1A). The airway volume, which ranged from -1024 to -500 Hounsfield units (HU), was calculated automatically with the software using the multiplanar volume-rendering method (Fig 1B).

In addition, the mandible was also segmented from a CT-value range of 200–4000 HU, and the area of intra-mandibular space was calculated to normalize tongue volume, because the mandible is an enclosed structure that defines the relative size of the tongue. As previously reported [26], we defined the most anterior-inferior point of the mandible (gnathion) and the most posterior-inferior points of the mandible (right and left gonion). Both mandibular body lengths were measured as the distance from the gnathion to both gonion, and mandibular width was defined as the distance between both gonions. Mandibular depth was calculated as the distance from the gnathion to the midpoint between both gonions. The mandibular angle was defined as the angle between the extension lines of right and left mandibular body length. The inner mandibular area (IMA) was calculated using mandibular lengths and the mandibular angle (Fig 1C and 1D).

### Statistical analysis

The association of various factors (AHI, RDI, lowest O2 saturation, ODI, tongue volume, corrected tongue volume, PASV, Mallampati score, Friedman stage, PAS, MP-H, BMI) was analysed using univariate and multivariate linear regression tests. Factors with a p-value less than 0.05 in the univariate analysis were further tested by multivariate logistic regression analyses. Adjusted odds ratios (95% confidence intervals) were calculated for independent variables (tongue volume, posterior airway space volume, and tongue volume/mandibular area). Pearson correlation coefficients were also tested between variables and polysomnographic findings. Parametric distribution was determined by Kolmogorov-Smirnov test. The normal-mild and moderate-severe OSA groups were compared by *t*-test. Statistical analyses were performed with SPSS version 2.0 (IBM Corporation, Armonk, NY, USA).

## Results

### Subject profiles

Sixty-four male patients with normal to severe OSA were analysed, and their mean age was 43.8 ± 12.3 years. They were divided into the following four groups, according to their AHI to show their profiles: normal; AHI 0~4, mild; AHI 5~14, moderate; AHI 15~29, and severe; AHI 30~. The numbers of patients, age, and the AHI in each group were as follows: normal (n = 9, 42.2 ± 10.5 years, AHI = 2.2 ± 1.4), mild (n = 13, 44.6 ± 13.2 years, AHI = 9.9 ± 2.8), moderate (n = 6, 40.8 ± 18.2 years, AHI = 21.4 ± 4.6), and severe (n = 36, 44.3 ± 11.7 years, AHI = 58.4 ± 17.9).

### Association between volumes and polysomnographic or physical parameters

**Fig 3:**
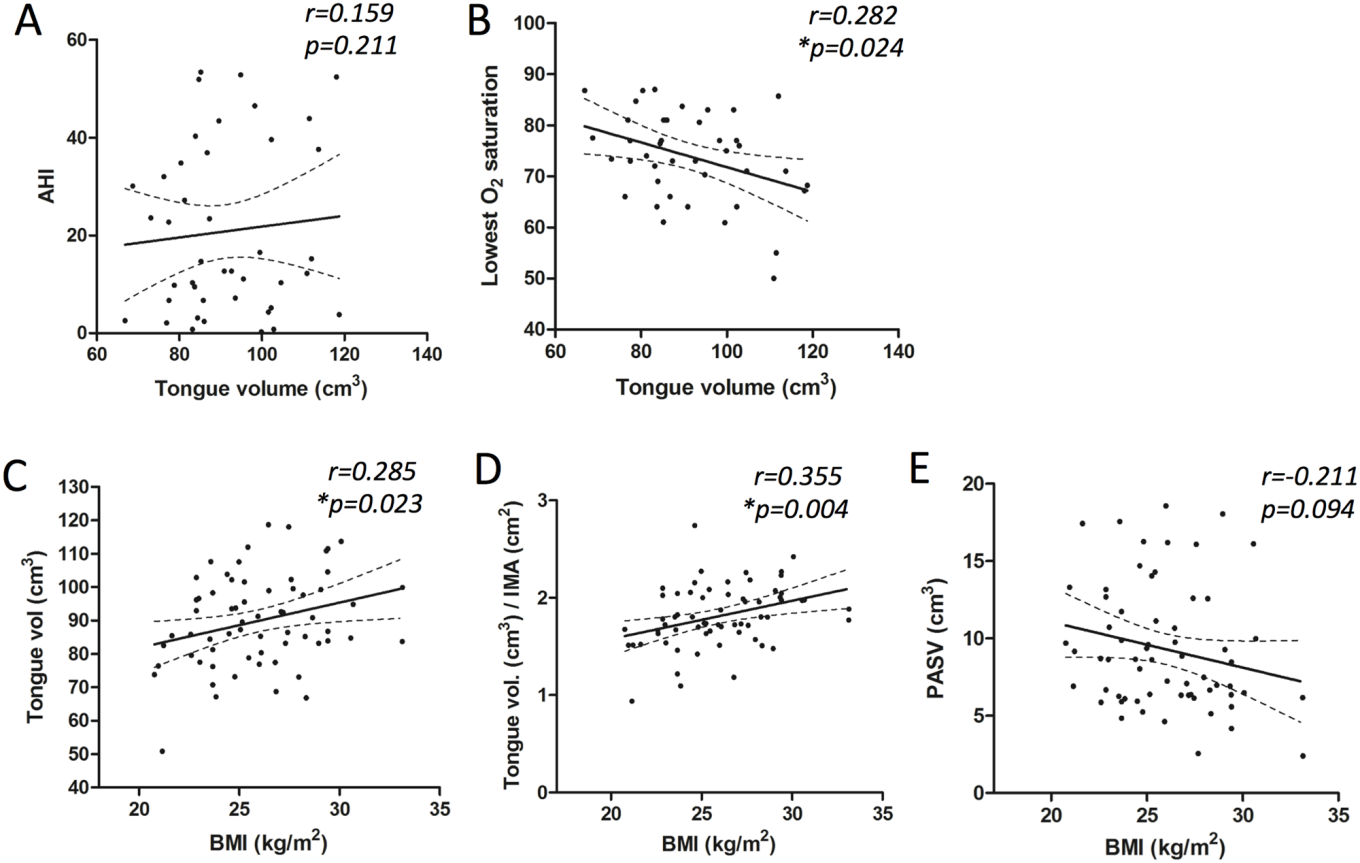
Linear regression analysis between tongue volume and polysomnographic parameters (A and B) and between BMI and tongue volume or PASV (C~E). Line indicates mean with 95% CI. AHI; apnea-hypopnea index, BMI; body mass index, PASV; posterior airway space volume

The relative tongue volume (tongue vol. / IMA) and posterior airway space volume (PASV) had no correlation with polysomnographic parameters, such as the AHI (Fig 3A), RDI, or lowest O_2_ saturation. Interestingly, only absolute tongue volume showed significant association with lowest O_2_ saturation (r = -0.282, p = 0.024, Fig 3B, Table 1). Physical parameters, such as the modified Mallampati grade, Friedman score, PAS, MP-H, and BMI were also determined. The modified Mallampati grade showed significant correlation with the AHI (r = 0.295, p = 0.018) and RDI (r = 0.318, p = 0.010). However, Freidman stage was not associated with polysomnographic parameters. The MP-H was strongly associated with the AHI (r = 0.405, p = 0.001) and RDI (r = 0.376, p = 0.002). In addition, BMI had strong positive correlation with AHI (r = 0.474, p<0.001) and RDI (r = 0.517, p<0.001). A negative correlation was found between with lowest O_2_ saturation and BMI (r = -0.532, p<0.001). Therefore, we investigated the influence of BMI on absolute and standardized tongue volume and PASV (Fig 3C, 3D and 3E). Interestingly, high tongue volume was associated with obesity.

**Table 1:** Correlation between volumetric parameters and physical findings or polysomnographic features.

	AHI		RDI		Lowest O_2_ sat		ODI	
	r	p-value	r	p-value	r	p-value	r	p-value
Tongue vol.	0.159	0.211	0.181	0.152	-0.282	0.024*	0.124	0.329
Tongue vol. / Intramandibular area	0.124	0.331	0.138	0.275	-0.120	0.346	0.034	0.791
PASV	-0.080	0.530	-0.112	0.379	0.205	0.104	-0.127	0.316
Mallampati	0.295	0.018*	0.318	0.010*	-0.211	0.094	0.262	0.036*
Friedman	-0.126	0.323	-0.120	0.343	0.047	0.713	-0.096	0.448
PAS	0.079	0.534	0.129	0.310	-0.078	0.539	-0.078	0.539
MP-H	0.405	0.001**	0.376	0.002**	-0.191	0.130	0.374	0.002**
BMI	0.474	<0.001**	0.517	<0.001**	-0.532	<0.001**	0.514	<0.001**

Abbreviations: AHI, apnea-hypopnea index; BMI, body mass index; PAS, posterior airway space; PASV, posterior airway space volume; MP-H, mandibular plane-to-hyoid distance; vol, volume; ODI, oxygen desaturation index; RDI, respiratory disturbance index; sat, saturation;

*, p<0.05;

**, p<0.005

Multivariate regression analyses were performed to further investigate important volumetric parameters that might affect polysomnographic parameters. Similarly, absolute tongue volume was not associated with AHI or RDI, but significantly associated with lowest O_2_ saturation (p = 0.027, Table 2).

**Table 2:** Association of the AHI, RDI, or lowest O_2_ saturation level with volumetric variables by multivariate linear regression analyses.

	Variables	B ± SE	p-value
AHI	Tongue vol.	0.280 ± 0.294	0.345
	PASV	-0.652 ± 0.878	0.460
	Tongue vol./Intramandibular area	5.230 ± 11.706	0.657
RDI	Tongue vol.	0.307 ± 0.273	0.266
	PASV	-0.832 ± 0.817	0.312
	Tongue vol./ Intramandibular area	5.338 ± 10.890	0.626
Lowest O_2_ sat	Tongue vol.	-0.230 ± 0.102	0.027*
	PASV	0.582 ± 0.304	0.060
	Tongue vol./ Intramandibular area	0.416 ± 4.053	0.919
ODI	Tongue vol.	0.305 ± 0.293	0.302
	PASV	-0.955 ± 0.877	0.280
	Tongue vol./ Intramandibular area	-2.431 ± 11.687	0.836

Abbreviations: AHI, apnea-hypopnea index; B, regression coefficients; BMI, body mass index; ODI, oxygen desaturation index; PASV, posterior airway space volume; vol, volume; SE, standard error; sat, saturation;

*, p<0.05

**Fig 4:**
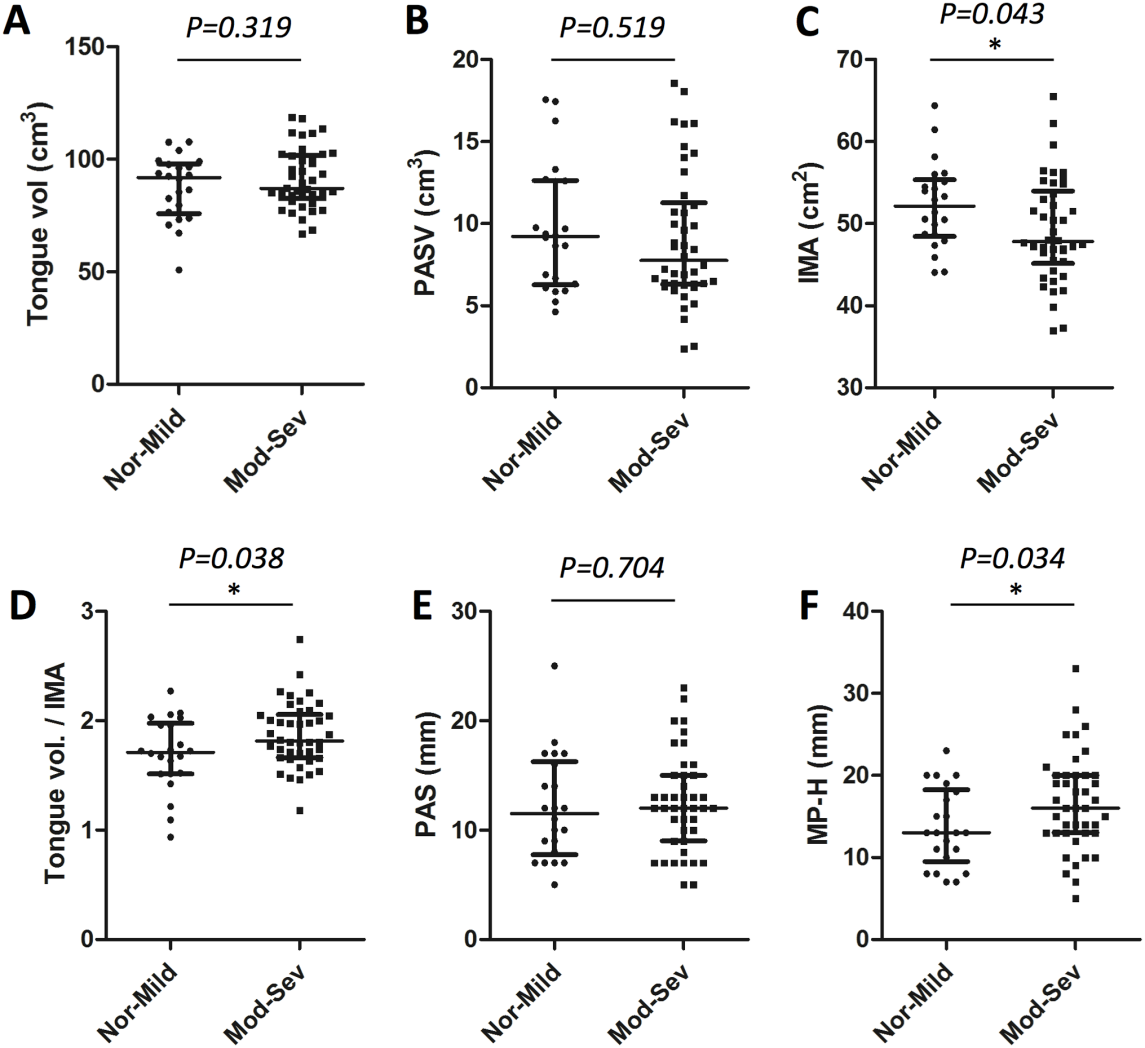
Scatter dot plots and comparisons of various measurements between normal-mild group and moderate-severe group. Line indicates median with interquartile range. IMA; inner mandibular area, MP-H; mandibular plane-to-hyoid distance, Mod; moderate, Nor; normal, PAS; posterior airway space, PASV; posterior airway space volume, Sev; severe

All patients were then re-grouped into normal-mild OSA (Nor-Mild) and moderate-severe OSA (Mod-Sev) groups categorized by AHI and compared by t-test (Table 3, Fig 4). Among the volumetric parameters, standardized tongue volume (Tongue vol./IMA) was significantly greater in the Mod-Sev group than in the Nor-Mild group (p = 0.038). The IMA was also smaller in the Mod-Sev group compared to Nor-Mild group (p = 0.043). The absolute tongue volume and PASV were not different between two groups as similar to our multivariate regression result. Furthermore, the MP-H was significantly longer in the Mod-Sev group than in the Nor-Mild group (p = 0.034).

**Table 3:** Comparison of volumes and physical findings between normal-mild and moderate-severe obstructive sleep apnea patients.

	Normal-Mild	Mod-Severe	P-value
AHI	6.8 ± 4.5	53.1 ± 21.1	
RDI	11.2 ± 6.3	54.9 ± 19.4	
Lowest O_2_ sat (%)	88.4 ± 4.4	73.9 ± 8.8	< 0.0001**
Subject number	22	42	
Age (yr)	43.6 ± 12.0	43.8 ± 12.6	0.957
BMI (kg/m^2^)	24.2 ± 2.4	26.9 ± 2.7	< 0.001**
Tongue vol. (cm^3^)	87.5 ± 14.4	91.2 ± 13.3	0.319
PASV (cm^3^)	9.8 ± 3.9	9.1 ± 4.1	0.519
Intramandibular area (cm^2^)	52.2 ± 5.2	49.2 ± 6.3	0.043*
Tongue vol./Intramandibular area	1.69 ± 0.3	1.87 ± 0.3	0.038*
Tonsil grade	1.5 ± 0.6	2.0 ± 0.9	0.033*
Mallampati grade	2.5 ± 1.0	2.8 ± 0.8	0.189
Friedman stage	2.5 ± 0.5	2.4 ± 0.7	0.429
Cephalometry lateral			
PAS (mm)	12.0 ± 4.9	12.5 ± 4.5	0.704
MP-H (mm)	13.7 ± 4.8	16.7 ± 5.9	0.034*

Abbreviations: AHI, apnea-hypopnea index; PAS, posterior airway space; PASV, posterior airway space volume; MP-H, mandibular plane-to-hyoid distance; mod-severe, moderate-severe;

*, p<0.05;

**, p<0.005.

### Correlation between physical findings and polysomnographic parameters

**Fig 5:**
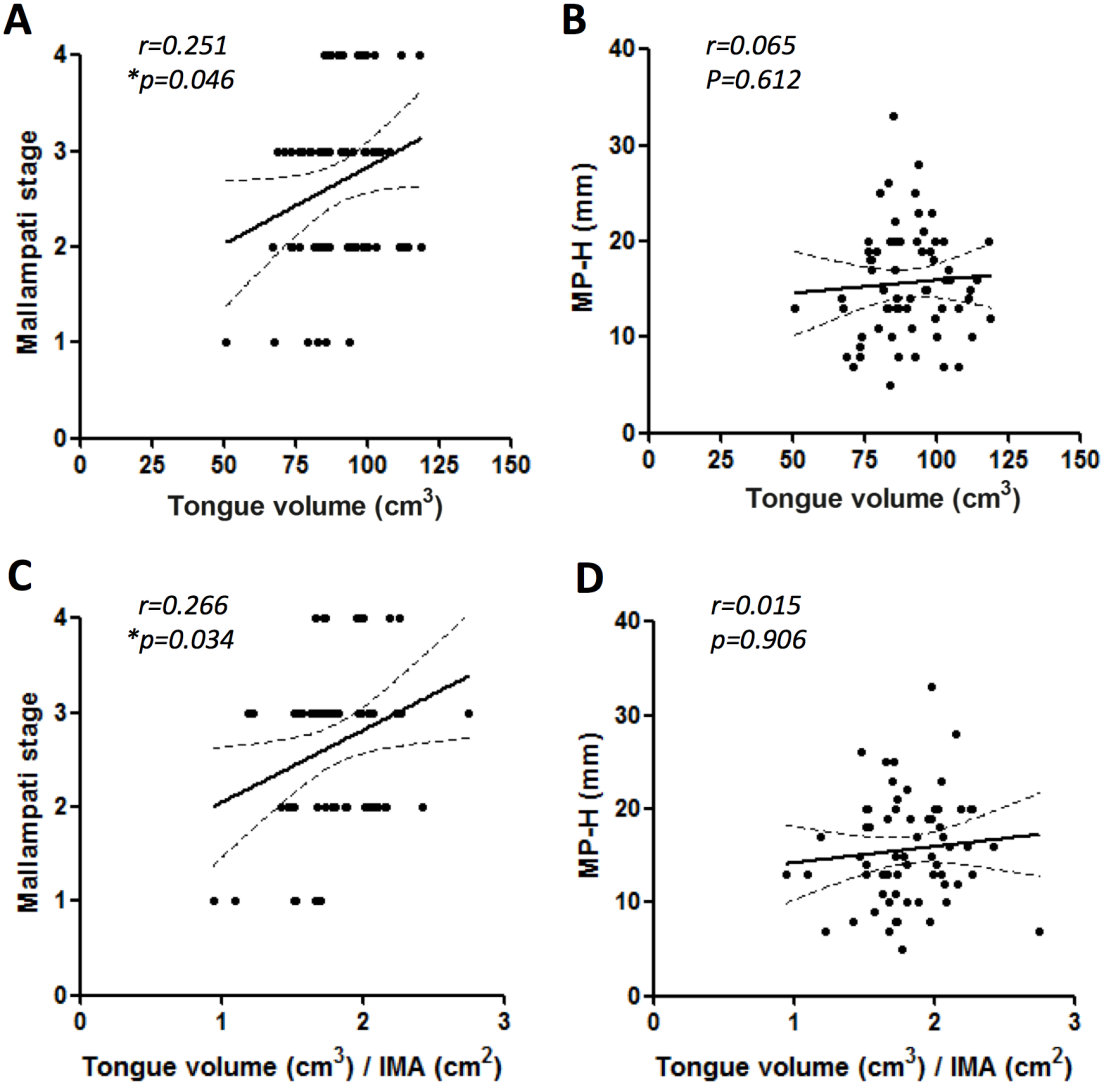
Correlation between tongue volume and the (A) Mallampati stage by physical examination or (B) mandibular plane-to-hyoid distance (MP-H). Correlation between standardized tongue volume (absolute tongue volume / intramandibular area) and the (C) Mallampati stage or (D) MP-H.

Our findings show that tongue volume was an important factor that strongly correlated with lowest O_2_ saturation during sleep in OSA patients. Therefore, physical factors were investigated to predict the size of the tongue that may affect lowest O_2_ saturation. Because the univariate analysis identified both the Mallampati stage and MP-H as meaningful factors in all subjects, these two parameters were further analysed by the multivariate test (Table 4). Interestingly, the Mallampati stage, and not the MP-H, exhibited higher association with tongue size (p = 0.04, Fig 5).

**Table 4:** Association of tongue volume with the Mallampati stage or MP-H by univariate and multivariate linear regression analyses.

		Univariate analysis		Multivariate analysis	
	Variables	B ± SE	p-value	B ± SE	p-value
Tongue vol.	Mallampati stage	3.943 ± 1.933	0.046	3.921 ± 1.945	0.048
	MP-H	0.157 ± 0.307	0.612	0.142 ± 0.300	0.637
Tongue vol. / Intramandibular area	Mallampati stage	0.105 ± 0.048	0.034*	0.104 ± 0.049	0.035*
	MP-H	0.001 ± 0.008	0.906	0.001 ± 0.007	0.944

Abbreviations: B, regression coefficients; SE, standard error; MP-H, mandibular plane-to-hyoid distance;

*, p<0.05

## Discussion

The tongue is a critical anatomical structure in OSA patients. Therefore, the evaluation of the tongue is essential to predict the severity and obstruction level of OSA. Tongue volume is correlated with obesity, and OSA patients with higher BMI are likely to have greater tongue size [16, 21]. Brennick et al. reported that excess fat is deposited in the tongues of obese mice, thus causing airway compromise [27]. Because high BMI is closely related to the occurrence of sleep apnea, tongue volume may be associated with the severity of OSA by enhancing the collapsibility of the retroglossal region. There are known differences in craniofacial configuration between Caucasian and Asian OSA patients. Asian patients have a greater tendency to exhibit craniofacial bony restriction than Caucasian patients [28]. Hence, the size of the oral cavity, as defined by the mandible, should be considered. In this study, we measured tongue volume in 3-D-reconstructed MDCT images and evaluated standardized tongue volume, which was normalized by the inner mandible area (tongue volume/IMA). Interestingly, absolute tongue volume was not correlated with the AHI or RDI, but associated with lowest O_2_ saturation (Tables 1 and 2), and this was similar to results that have been reported in other studies with Asian patients [16, 21]. However, standardized tongue volume, which implies mandible size, was much higher in Mod-Sev OSA group and this indicates that the craniofacial bony frame is a relevant factor that might aggravate the severity of AHI in OSA patients (Table 3). This supports the previous findings using 2D measurements [29]. Therefore the absolute tongue volume is also affected by BMI in Asian race and this may be an important factor for lowest O_2_ saturation, but small mandible size is more related to AHI. Therefore, the estimation of tongue volume by 3D imaging analysis or physical examination can be very useful in determining surgical procedures or predicting surgical outcomes.

The physical examination of the oral cavity is crucial for finding anatomical abnormalities. The modified Mallampati grading, as suggested by Friedman et al.[24], is a simple method to estimate the relationship between the tongue and soft palate. The advantage of the grading method is that it can provide the usual tongue position, which is similar to the position of the tongue during sleep. Furthermore, it is a reliable predictor of OSA, and it significantly correlates with the RDI. In this study, we showed that a high modified Mallampati score was meaningfully associated with absolute tongue volume. Tongue volume is known to be the pathogenic factor that contributes to the inferior-posterior positioning of the tongue, thus increasing the MP-H [19]. However, in this study, there was no positive correlation between tongue volume and MP-H by cephalometric analysis. Cephalometry is a standard method that is widely used to determine various structures in OSA patients. In general, the inferiorly positioned hyoid bone has been reported by many researchers to be one of many predictors for OSA [19, 30]. We also observed a strong positive correlation between the MP-H and the severity of the AHI (Table 1), and moderate-severe OSA patients were likely to have a longer MP-H than normal-mild OSA patients (Table 3). A significantly positive correlation was observed between tongue volume and the modified Mallampati grade. However, in contrast to a previous study [19], no association between tongue volume and the MP-H was found. This suggests that, instead of the MP-H, the modified Mallampati grade can be used to predict absolute tongue volume.

The upper airway space volume is closely related to obesity, and weight loss can increase the luminal volume of the upper airway space by reducing the size of the parapharyngeal fat pad [17]. Schwab et al. reported that the retropalatal airway volume is smaller in apneic patients than in normal subjects. Shigeta et al. observed that the airway space volume decreases when BMI increases, but it was not associated with the severity of the AHI [16]. Our results show that the upper airway space volume had no significant influence on the severity of the AHI or lowest O_2_ saturation (Tables 1, 2 and 3). These discrepancies among several studies may be due to differences in subjects’ ethnicities or measurement methods. The enlargement of airway space volume by various treatments, including weight loss, is still very important for improving or treating sleep apnea. However, creating a larger airway space by surgery may not be enough to decrease the AHI, and any surgical techniques that can increase tension on the pharyngeal wall may have a better impact on successful outcomes. This is demonstrated by low success rates following conventional uvulopalatopharyngoplasty, which simply enlarges the pharyngeal space. However, surgeries that reduce tongue volume may improve lowest O_2_ saturation, which has a beneficial influence on the comorbidity of sleep apnea. In addition, OSA patients with hypertension tend to have lower O_2_ saturation nadir during sleep than OSA patients without hypertension [31]. Interestingly, positive results have been reported in patients undergoing the combination of transoral robotic surgery for tongue base resection and expansion sphincter pharyngoplasty, which may indicate the importance of palatal surgeries that augment tension and resect the base of the tongue [32].

This study has some limitations. First, the changes of tongue or airway shape during sleep would be more critical, but CT scans in this study were taken during wakefulness. Second, CT scan does pose a risk of radiation exposure, which must be considered for routine use, although CT scan has several advantages over MRI. Third, a small number of normal subjects was included, and we re-grouped all subjects into two groups (normal-mild vs. moderate-severe) to compare their features based on AHI severity. Further studies that enroll a larger number of normal patients will sharpen our results. Nevertheless, as strengths of our study, our study population comprised a single Asian ethnic group (Korean), and only male patients, who were mostly middle-aged, were included. In addition, a CT scan was recommended and undertaken after obtaining permission from all patients screened for OSA that visited our sleep clinic to investigate anatomical issues from the nose to the neck. Therefore we believe that this may possibly minimize any selection bias that may occur due to racial and gender differences. Furthermore, we utilized high-resolution MDCT, which is a reliable tool for examining OSA patients.

In this study, we were unable to provide the exact reason for why tongue volume is closely associated with lowest O_2_ saturation (not AHI). However, we suggest two possible explanations. First, tongue volume may be a surrogate marker for the influence of obesity on O_2_ saturation through lung volume changes. In our results shown in Table 2, the BMI was not included as a covariate in the multivariate analysis, as BMI is significantly associated with tongue volume and using BMI as a covariate in the multivariate analysis revealed no association with tongue volume. Second, the AHI is an essential factor in diagnosis and predicting prognosis; however, it is not a perfect index. The AHI is calculated as the sum of apnea and hypopnea events per hour. Therefore, it can represent sleep fragmentation by frequent arousal very well, although longer-lasting durations of apnea and hypopnea and the severity of hypoxemia may be inaccurately conveyed. We observed that the longest apnea time was significantly associated with the lowest O_2_ saturation on multivariate analysis (r = 0.726, p<0.001; S1 Table) and previously reported that the lower O_2_ saturation nadir may be an important value that should be considered [31]. In the future, an index more precise than AHI that can predict the severity of OSA and its prognosis should be developed for better management of OSA patients.

This study was conducted in an attempt to outline the relationships between tongue or airway space volume measured from 3-D-reconstructed MDCT scans and polysomnographic parameters in Korean male OSA patients to provide useful insights for the surgical treatment thereof. In addition, the relationship between tongue volume and the modified Mallampati grading method was also investigated and showed a strong association. The absolute tongue size was more associated with lowest O_2_ saturation than the severity of AHI and the obesity was a relevant factor for an increase in absolute tongue volume. Therefore for those who have high BMI and modified Mallampati grade in Asian OSA patients, the reduction of isolated tongue volume would be not enough to decrease AHI, but may have beneficial effect to improve the lowest O_2_ saturation that is associated with comorbid hypertension in OSA and to reduce cardiovascular morbidity.

## Supporting Information

S1 Table Association of lowest O_2_ saturation with the apnea or hypopnea duration by univariate and multivariate linear regression analyses. (DOCX)
