# Supplementary material for: Tongue Volume Influences Lowest Oxygen Saturation but Not Apnea-Hypopnea Index in Obstructive Sleep Apnea
Source: PLoS One. 2015 Aug 17;10(8):e0135796. doi: 10.1371/journal.pone.0135796 (PMC4539216; doi:10.1371/journal.pone.0135796)
Supplement: S1 Table — (DOCX) [file pone.0135796.s001.docx]

**S1 Table.** Association of lowest O_2_ saturation with the apnea or hypopnea duration by univariate and multivariate linear regression analyses

|  |  | Univariate analysis | | Multivariate analysis | |
| --- | --- | --- | --- | --- | --- |
|  | Variables (sec) | B ± SE | p-value | B ± SE | p-value |
| Lowest O_2_ sat | Longest Apnea duration | -0.331 ± 0.041 | < 0.001* | -0.325 ± 0.078 | < 0.001* |
|  | Mean Apnea duration | -0.633 ± 0.108 | < 0.001* | -0.043 ± 0.175 | 0.808 |
|  | Longest Hypopnea duration | -0.269 ± 0.080 | 0.001* | -0.078 ± 0.084 | 0.358 |
|  | Mean Hypopnea duration | -0.471 ± 0.258 | 0.072 | 0.375 ± 0.180 | 0.132 |

Abbreviations: B, regression coefficients; SE, standard error; *, p<0.05
